# Clinical presentation and prognostic indicators in 100 adults and children with neurofibromatosis 1 associated non-optic pathway brain gliomas

**DOI:** 10.1007/s11060-017-2475-z

**Published:** 2017-06-07

**Authors:** Susan Byrne, Steve Connor, Karine Lascelles, Ata Siddiqui, Darren Hargrave, Rosalie E. Ferner

**Affiliations:** 1 National Neurofibromatosis Service, Guy’s and St. Thomas’ NHS Foundation Trust, London, UK; 2 Department of Neurology, Guy’s and St. Thomas’ NHS Foundation Trust, London, UK; 3 Department of Paediatric Neurology, Evelina Children’s Hospital, Guy’s and St. Thomas’ NHS Foundation Trust, London, UK; 4 Institute of Psychiatry, Psychology, and Neuroscience, Kings College London, London, UK; 5 Department of Neuroradiology, Guy’s and St. Thomas’ NHS Foundation Trust, London, UK; 6 Department of Neuroradiology, King’s College Hospital NHS Foundation Trust, London, UK; 7 Department of Paediatric Oncology, Great Ormond Street Hospital, London, UK

**Keywords:** Neurofibromatosis type 1 (NF1), Brain tumour, Pilocytic astrocytoma, Epidemiology, Neurooncology, Thalamic tumour

## Abstract

Type 1 Neurofibromatosis (NF1) is a common autosomal dominant condition, with a major impact on the nervous system, eye, bone, and skin, and a predisposition to malignancy. At present it is not possible to predict clinically or on imaging, whether a brain tumour will remain indolent or undergo high-grade change. There are no consensus guidelines on the follow-up of non-optic pathway glioma (non-OPG) tumours in NF1. One hundred patients from the National NF1 Service with generalised NF1 and a diagnosis of non-OPG glioma were followed up for a median time of 63 months after glioma detection. Forty-two patients underwent surgical intervention. Ninety-one percent (38) of those requiring surgery did so within 5 years of diagnosis of glioma. Serial neuroimaging was undertaken in 88 patients. In 66 (75%), the lesion on the scan was stable or had improved at follow-up. High-grade lesions were present in five patients and were strongly associated with tumours in the thalamus (p = 0.001). Five patients died during follow-up. The diagnosis of high-grade glioma had a HR of 99.7 (95% CI 11.1–898.9, p < 000.1) on multivariate Cox regression to evaluate predictive factors related to death. In our cohort of 100 patients with NF1, we have shown that tumours in the thalamus are more likely to be associated with radiological progression, high-grade tumours, and surgical intervention. As a result of this finding, heightened surveillance with more frequent imaging should be considered in thalamic involvement. We have also demonstrated that over 40% of patients underwent surgery, and did so within 5 years of tumour diagnosis. Serial imaging should be undertaken for at the very least, 5 years from tumour detection.

**Electronic supplementary material:**

The online version of this article (doi:10.1007/s11060-017-2475-z) contains supplementary material, which is available to authorized users.

## Background and aims

Type 1 Neurofibromatosis (NF1) is a common autosomal dominant condition, with a major impact on the nervous system, eye, bone, and skin. NF1 is a tumour suppressor disorder that is typified by benign nerve sheath tumours and a predisposition to malignancy. Lack of the *NF1* gene product neurofibromin arising from gene mutation causes activation of the ras pathway and unrestrained cell growth [1, 2]. The natural history of NF1-associated tumours differs from the general population [3], and NF1 individuals require specialist assessment and management.

Low-grade brain gliomas are a well-recognized complication of NF1 and they may remain indolent over many years; however, a small number of low-grade gliomas transform into more aggressive tumour types [4]. At present it is not possible to predict clinically or on imaging, whether a tumour will remain indolent or undergo high-grade change. Another small subset of patients may present acutely with a high-grade brain tumour without ever having had a low-grade glioma identified. A study by Guillamo et al. looked at brain gliomas in NF1 and identified that extra-optic location, tumours diagnosed in adulthood, and symptoms at diagnosis are associated with a higher mortality [4].

Two decades ago it was common practice to treat these low-grade tumours aggressively, however, more recent epidemiological studies have demonstrated an association between radiotherapy and subsequent malignant transformation [3], endocrine sequelae, neurovascular, and cognitive difficulties. The majority of non-optic pathway glioma in NF1 are indolent, low-grade pilocytic astrocytomas and become symptomatic only when there is mechanical obstruction, or less commonly when malignant changes occur [5].

At the current time there are no consistent guidelines for identifying or monitoring non-optic pathway gliomas (non-OPG) in patients with NF1, and this reflects the paucity of information on natural progression of these tumours.

The aim of our study was to carry out a retrospective review of all patients who attended our national neurofibromatosis centre between 2009 and 2014 and who had a non-OPG glioma. The goal was to characterise the clinical presentation, management, progression, and outcome in this cohort.

## Methods

All patients attending the National NF1 Service from 2009 to 2014 with generalised NF1 (diagnosis made by clinical criteria and genetic analysis where appropriate) and ever had a history of non-OPG glioma were included. Data were extracted from clinical records by two researchers (SB, CW). Brain glioma was diagnosed on histology and/or suspected on neuroimaging by two neuroradiologists with extensive experience in NF1 (over 1000 patients in 15 years) using criteria such as persistent lesional enhancement and/or mass effect. The neuroradiologists compared follow-up imaging to previous imaging using linear measurement and qualitative assessment. Patients with mosaic NF1 or with brain metastases were excluded from assessment. Reason for imaging was determined as (1) symptomatic—if the scan was carried out for symptoms related to the anatomical location of the tumour (2) unrelated—if the MRI was undertaken by the referring institution for screening purposes (although this is not our practice) or for symptoms unrelated to anatomical location of the tumour.

Extended disability scores (EDSS) [6] were calculated using reported examination at each visit. Information collected is outlined in supplementary table 1.

Data analysis was carried out using SPSS 22 [IBM SPSS Statistics for Macintosh, Version 22.0]. Parametric tests were carried out where the data were normally distributed, and non-parametric tests were used if the data were not normally distributed. Chi Square testing or Fisher’s exact testing was used to compare two the relationship between two categorical variables. Survival analysis, and time to event analysis was carried out using univariate and Cox multivariate survival analysis, with time variable being time from glioma diagnosis to the event in question (e.g. surgery, death) or censor date. Statistical significance was set at <0.05.

This study was approved as a clinical evaluation with study number 5099 by the Clinical Audit Group Committee at Guy’s and St. Thomas’ NHS Foundation Trust, London.

## Results

One hundred patients from our cohort had generalised NF1 and a diagnosis of non-OPG glioma (47 males, 53 females). Median age at glioma diagnosis was 16 years (range 2–62 years), and 57 patients were under the age of 18 years at diagnosis. Patients were followed up for a median time of 63 months (range 0–452 months) after a glioma was detected.

Neuroimaging was initially performed in 44/100 patients with glioma-related symptoms, in 53/100 individuals for unrelated reasons, and the reason was not available in 3/100 (see supplementary table 2).

**Fig. 1:**
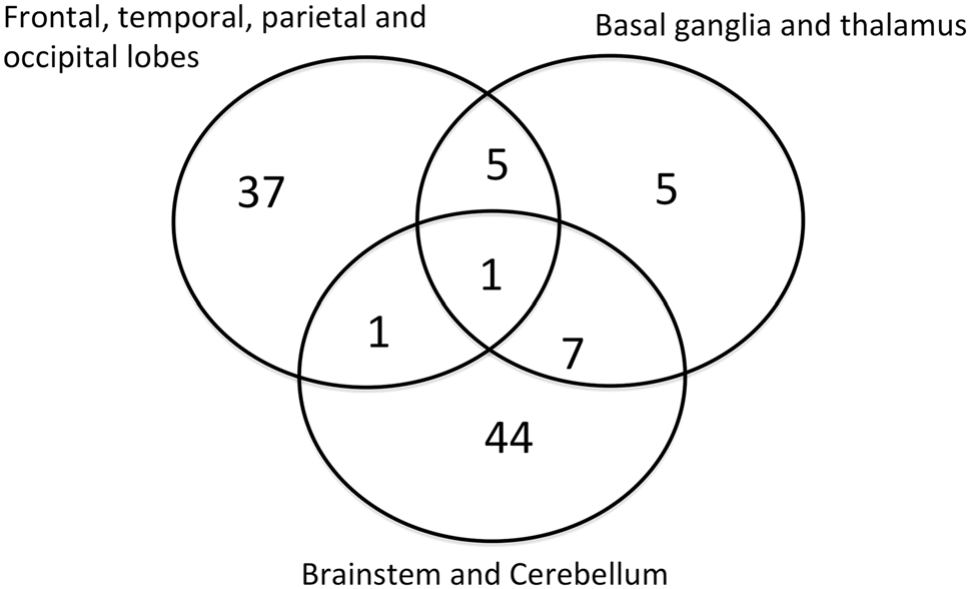
Venn diagram of location of non-OPG (n = 100)

Location of tumours can be seen in Fig. 1. Diagnosis was made on neuroimaging only in 58/100, and on histological analysis and neuroimaging in 42/100.

### Surgical intervention

Forty-two of the 100 patients underwent surgical intervention during follow-up; initial surgical intervention was total or subtotal resection of tumour in 29, biopsy only in 6, and shunt or ventriculostomy only in 7 individuals.

Twenty-four patients had surgery for relief of symptoms, ten patients had surgery because of concern regarding the radiological features of the tumour, and in eight cases the specific reason for surgery was not stated in the medical notes.

Ten patients went on to have further surgical intervention at a later stage. Of the ten who had further surgery, five required debulking (initial surgery was debulking in three, initial surgery was shunt/ventriculostomy in two), three required shunt revision (initial surgery was ventriculostomy/shunt for all three), one required a shunt (initial surgery was a biopsy), and one required a shunt and Omaya reservoir insertion (initial surgery was a biopsy).

The requirement for surgical intervention was much higher in the cohort of patients who had a scan because of glioma-related symptoms at presentation (31 of 44; 71%), compared to those who had an MRI for screening or other reasons (11 of 56), p < 0.0001. Symptomatic reasons for surgical intervention included seizures, raised intracranial pressure, rapid increase in size of tumour, and hemiparesis.

**Fig. 2:**
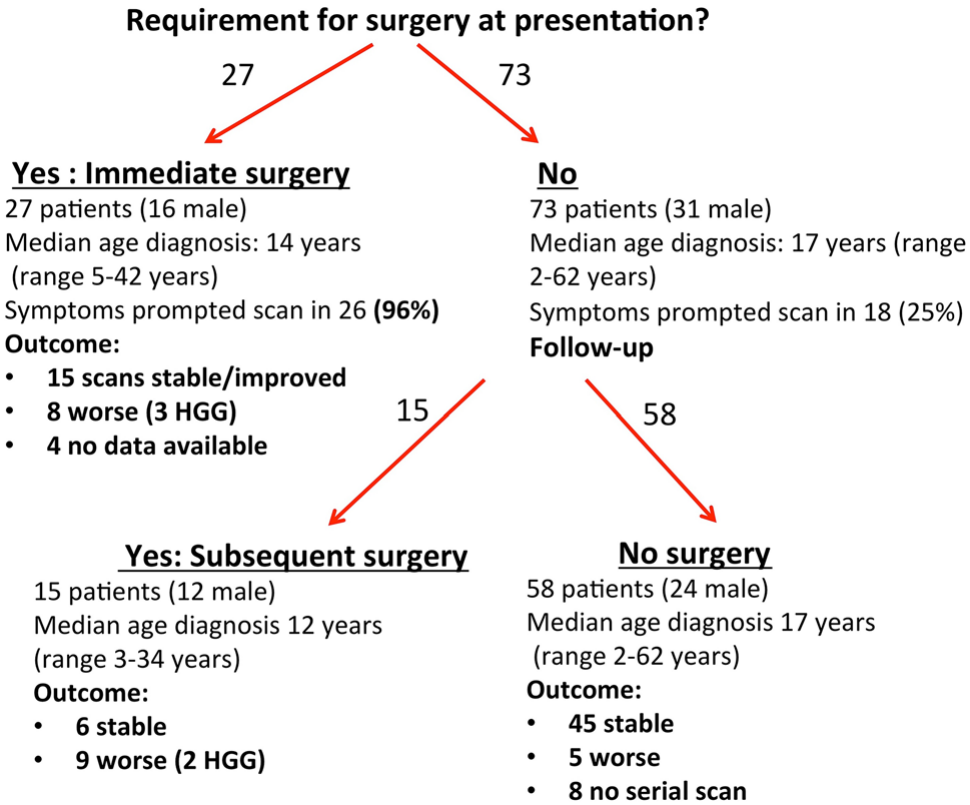
Flow diagram of the requirement for surgery in 100 patients with NF1 related glioma (immediate surgery refers to surgery within 2 months of initial presentation, *HGG* high grade glioma)

Twenty-seven of 42 patients (64%) requiring surgical intervention did so within 2 months of presentation, and 38/42 (91%) had surgery within 5 years of diagnosis of glioma. Only four patients (9%) had surgery beyond 5 years, three because of radiological progression and one because of symptoms (see Fig. 2).

Multivariate Cox regression was performed looking at predictive factors for surgery. Significant variables were thalamic involvement [Hazard ratio (HR) 2.18, 95% CI 1.01–4.6, p = 0.049], frontal involvement (HR 3.36, 95% CI 1.49–7.6, p = 0.004), and cerebellar involvement (HR 4.44, 95% CI 2.0–9.8, p < 0.0001). Presence of a high-grade glioma was also a significant risk factor for surgery (HR 3.46, 95% CI 1.33–9, p = 0.011). Age at diagnosis was not a significant variable (HR 0.975, 95% CI 0.94–1.004, p = 0.087).

Three patients required surgical intervention, radiotherapy and chemotherapy, four had surgery and chemotherapy, and four had surgery and radiotherapy. None had chemotherapy alone. Of the 11 patients who received more than one treatment modality, eight patients had multimodal treatment initially, and three patients had additional treatments added after progression.

### Histology

Formal histology results were available on 32 patients: 23 pilocytic astrocytoma, 5 glioblastoma multiforme, 3 ganglioglioma, and 1 pilomyxoid glioma (grade 2). One of our patients underwent molecular analysis and had a BRAF V600E mutation.

High-grade lesions were present in five patients and were strongly associated with tumours in the thalamus compared with other regions (4/12 compared to 1/88; p = 0.001). Three patients with HGG presented acutely with symptoms; two with headache and signs of papilloedema, and one with a new cranial nerve palsy and ataxia. Only two of the patients with HGG had previous imaging, both of which showed lesions involving the thalamus—one of the patients had previous debulking of a frontal/thalamic lesion, and the histology showed a pilomyxoid astrocytoma before transforming to a HGG.

Details of the five patients with high-grade glioma are outlined in Table 1.

**Table 1:** Clinical presentation, histology, treatment and outcome in five NF1 patients with high-grade gliomas

Age glioma diagnosis	Reason for scan	Location	Intervention	Histology	Alive/time to death from diagnosis
28 years	Facial nerve palsy	Left medial frontal cortex, right mid thalamic, right superior colliculus	Craniotomy	Grade 2 pilomyxoid astrocytoma	Alive, 20 months after grade 4 GBM diagnosed
			Further surgery, temozolomide, radiotherapy	Grade 4 astrocytoma	
8 years	Baseline imaging	Initially asymmetrical thalamic myelination/possible pilocytic astrocytoma on imaging	Debulking surgery, temozolomide	Grade 4 GBM	Died, 9 months after grade 4 GBM diagnosed
	Headache (2 years later)	Bilateral thalamic grade 4 GBM on next scan			
17 years	Headaches with papilloedema	Right thalamic mass extending to midbrain and into ventricle	Subtotal resection, temozolomide, radiotherapy	Initial histology pilocytic astrocytoma with high proliferation index and atypical features, revised to grade 4 GBM	Died, 9 months after presentation
6 years	New onset ataxia with superior oblique palsy	Mass in left thalamus, caudate nucleus, cerebellum, brainstem	Empiric treatment with vincristine and carboplatin but biopsy when did not respond	Grade 4 GBM	Died, 6 months after presentation
32 years	Headaches, ataxia, seizure	Right cerebellar mass	Ventriculostomy, subsequent debulking, radiotherapy	Astrocytoma grade 3	Died, 16 months after presentation

### Survival analysis and outcome

Five of the 100 patients died during follow-up; two from progression of a low grade tumour to a high-grade glioma, two presented acutely with a high grade tumour in the first instance, and one patient developed cerebellar necrosis following radiotherapy. The survival rate from diagnosis was 95% at 5 years (95% CI 92.6–97.4%). On multivariate Cox regression to evaluate predictive factors related to death, the diagnosis of high grade glioma had a HR of 99.7 (95% CI 11.1–898.9, p < 000.1). There was no significant association between age at tumour detection and death.

At last follow-up 29/95 survivors had symptoms related to glioma with an EDSS of 2.2 (SD 1.6). Fifty-seven percent (21/37) of patients who had surgery had symptoms at follow up compared to 14% of patients who did not require surgery (p < 0.0001). Patients who were symptomatic at the time of diagnosis had a higher mean EDSS at most recent review (1.5) compared to individuals who had a glioma diagnosed incidentally (0.5), p = 0.01.

Serial neuroimaging was undertaken in 88 patients. The median time between first and most recent imaging was 59 months (range 2–453). In 66/88 (75%) the lesion on the scan was stable or had improved, and 22/88 (25%) showed radiological progression. Lesions in the thalamus (p = 0.004) were more likely to undergo radiological progression, however this was related to the transformation to high grade malignancy.

Previous history of OPG was present in 25/100 but was not associated with poorer outcome or high-grade tumour.

## Discussion

We have shown that tumours in the thalamus are more likely to be associated with high-grade tumours in NF1. As a result of this finding, heightened surveillance with more frequent imaging should be considered in patients with NF1 who have thalamic involvement. Individuals with tumours in the thalamus, frontal lobes, and cerebellum were more likely to require surgical intervention than patients with tumours in other anatomical locations. At the current time there are no consensus guidelines, and heightened surveillance in this subset of patients may lead to a decrease in morbidity and mortality.

Forty-two percent of patients (n = 42) underwent surgical intervention. Predictors of the requirement for surgical intervention included symptoms that necessitated cranial imaging, and thalamic, cerebellar and frontal lesions. We identified that 64% of patients requiring intervention had surgery within 2 months, and 91% of patients who needed intervention had surgery within 5 years of tumour detection. Overall the outcome was very favourable with 95% survival at 5 years, and 92% at 10 years. 65% of patients were asymptomatic at follow-up. We did not find an association between the age at tumour diagnosis and need for surgery and there was not association with death; however there was a trend towards significance and this may reflect the small size of out cohort.

The majority of tumours biopsied were pilocytic astrocytomas, and only five patients (5%) in this cohort developed a high-grade glioma. Our findings are supported by three studies [3, 4, 7] that reported that the majority of progressive tumours were not pilocytic astrocytomas but rather other types of astrocytoma, that are associated with a more aggressive histological subtype. Guillamo and colleagues showed that of 19 progressive tumours; only nine were pilocytic astrocytomas, with the remaining ten tumours comprising two low grade astrocytomas, two anaplastic astrocytomas, two glioblastomas, and one dysplastic neuroepithelial tumour [7].

Leonard and colleagues reported that over half of patients with unusual clinical presentation or radiographic features were not classic pilocytic astrocytomas on histological analysis, and highlighted the value of biopsy in those cases [8].

This study is limited by the fact that, histological diagnosis was only obtained in the minority of cases (32/100), with the remaining diagnoses were suspected radiologically. Radiological progression (improved/stable/worse) was assessed by experienced neuroradiologists, using linear measurement and qualitative assessment rather than volumetric analysis.

In NF1 individuals brain lesions that have irregular enhancement, have marked mass effect, or show restricted diffusion are probable gliomas that require judicious clinical and neuro-radiological follow up. There are currently no guidelines on the surveillance of NF1 patients once a non-optic pathway glioma has been identified. Lack of evidence makes determining a time interval for serial imaging extremely difficult, especially when patient anxiety is heightened by an underlying disorder that predisposes to tumours. We found that all high-grade gliomas and nearly all tumours requiring surgery did so within 5 years of tumour detection, and that the majority were symptomatic at presentation. Serial imaging should be undertaken for at the very least, 5 years from tumour detection.

We also found that tumours located in the thalamus were more likely to be associated with high-grade tumours (2 cases) and high-grade transformation (2 cases), so that location of tumour should direct one toward more frequent imaging. Krishnatry and colleagues reviewed LGG in over 1200 patients and found an association between mortality and thalamic lesion, however only 10% of this cohort had NF1 and the majority of patients with NF1 had an optic pathway glioma, and an excellent outcome [9]. As up to 85% of children with NF1 have abnormal signal on their MRI scans (FASIs), and the thalamus is a common location to see these changes [10], it is important to discuss cases with an experienced neuroradiologist to ensure that FASI don’t have any atypical features that may suggest the presence of a low grade glioma.

The patients in this series were referred to a national neurofibromatosis centre and may represent a more complex case mix, which may not be reflective of the NF1 population as a whole. However, from analysis of the data we found that over half of the gliomas were diagnosed when patients were scanned for another reason, and that fewer than one-fifth of patients identified in that way ever required surgery. These patients also had a better outcome in terms of EDSS at most recent review. It is difficult to tell if symptoms at follow up in those who had surgical interventions are secondary to tumour or to side effects of surgery. Our practice is to scan patients with clinical signs or symptoms and not to perform baseline neuroimaging, which may reveal incidental gliomas.

Under current WHO grading, brain tumours are given a Grade of I–IV depending on histological features [11]. In future the use of molecular genetic findings will aid in determining the course and prognosis of low-grade tumour types and also allow allocation of specific, targeted oncological therapy in these patients. One of our patients was diagnosed with a cerebellar ganglioglioma and had molecular genetic analysis that revealed a V600E BRAF mutation. The BRAF pathway is a key regulator of the MAPK pathway [12], and targeted treatment with a BRAF-MEK pathway inhibitor would be a potential therapy. Molecular genetic analysis of tumours may aid in decisions regarding therapy.

In conclusion, MRI should be undertaken in patients with NF1 who have signs and symptoms suggestive of glioma. Once a glioma is diagnosed any new symptoms indicating progression should be investigated promptly. Patient education regarding ‘red flag’ symptoms may hasten referral to neurosurgical services. Heightened surveillance with more frequent imaging should be considered in patients with thalamic involvement, and also in those with atypical histological findings for at least 5 years. Overall the outcome is very favourable with the majority of patients being symptom free at most recent follow-up.

## Electronic supplementary material

Below is the link to the electronic supplementary material.

Supplementary table 1. Information collected on each patient (DOCX 50 KB)

Supplementary table 2. Tumour related symptoms (n=44) (DOCX 54 KB)
