# Supplementary material for: Clinical presentation and prognostic indicators in 100 adults and children with neurofibromatosis 1 associated non-optic pathway brain gliomas
Source: J Neurooncol. 2017 Jun 7;133(3):609–14. doi: 10.1007/s11060-017-2475-z (PMC5537330; doi:10.1007/s11060-017-2475-z)
Supplement: Supplementary file 1 — Supplementary table 1. Information collected on each patient (DOCX 50 KB) [file 11060_2017_2475_MOESM1_ESM.docx]

| **Information recorded on each patient**  Demographics  Gender  Age  Family history of NF1  Genotype  NF1 and other co-morbidities  Date of presentation to NF1 service and diagnosis of glioma |
| --- |
| Clinical presentation  Method of diagnosis |
| Location of tumour |
| Need and reason for treatment |
| Type of treatment and number of interventions |
| Histology |
| EDSS |
| Current clinical status and symptoms related to glioma  Date and cause of death  MR Imaging  Number and timing of MRI |

*Supplementary table 1. Information collected on each patient*
