# Supplementary material for: Clinical presentation and prognostic indicators in 100 adults and children with neurofibromatosis 1 associated non-optic pathway brain gliomas
Source: J Neurooncol. 2017 Jun 7;133(3):609–14. doi: 10.1007/s11060-017-2475-z (PMC5537330; doi:10.1007/s11060-017-2475-z)
Supplement: Supplementary file 2 — Supplementary table 2. Tumour related symptoms (n=44) (DOCX 54 KB) [file 11060_2017_2475_MOESM2_ESM.docx]

| **Tumour related symptoms** | **Patients (%)** |
| --- | --- |
| Signs of hydrocephalus  (acute headache, ataxia, vomiting) | 12 (27%) |
| Focal seizure | 9 (21%) |
| Chronic headache (often with another feature; weight loss, abnormal clinical examination[e.g increased tone, asymmetric reflexes) | 6 (14%) |
| Hemiparesis | 5 (11%) |
| Sudden headache with change in vision | 4 (9%) |
| Diplopia | 2 (4%) |
| Other symptoms  -Co-ordination difficulties  -Torticollis  -Sensory change in limbs  -Swallowing problems  -Short stature | 6 (14%) |

*Supplementary table 2. Tumour related symptoms (n=44)*
